# Alterations in White Matter Fiber Tracts Characterized by Automated Fiber-Tract Quantification and Their Correlations With Cognitive Impairment in Neuromyelitis Optica Spectrum Disorder Patients

**DOI:** 10.3389/fnins.2022.904309

**Published:** 2022-07-01

**Authors:** Zichun Yan, Xiaohua Wang, Qiyuan Zhu, Zhuowei Shi, Xiaoya Chen, Yongliang Han, Qiao Zheng, Yiqiu Wei, Jingjie Wang, Yongmei Li

**Affiliations:** Department of Radiology, The First Affiliated Hospital of Chongqing Medical University, Chongqing, China

**Keywords:** neuromyelitis optica spectrum disorder, white matter, diffusion tensor imaging, automated fiber-tract quantification, cognitive impairment

## Abstract

**Objectives:**

To investigate whether patients with neuromyelitis optica spectrum disorder (NMOSD) have tract-specific alterations in the white matter (WM) and the correlations between the alterations and cognitive impairment.

**Materials and Methods:**

In total, 40 patients with NMOSD and 20 healthy controls (HCs) who underwent diffusion tensor imaging (DTI) scan and neuropsychological scale assessments were enrolled. Automated fiber-tract quantification (AFQ) was applied to identify and quantify 100 equally spaced nodes of 18 specific WM fiber tracts for each participant. Then the group comparisons in DTI metrics and correlations between different DTI metrics and neuropsychological scales were performed.

**Results:**

Regardless of the entire or pointwise level in WM fiber tracts, patients with NMOSD exhibited a decreased fractional anisotropy (FA) in the left inferior fronto-occipital fasciculus (L_IFOF) and widespread increased mean diffusion (MD), axial diffusivity (AD), and radial diffusivity (RD), especially for the thalamic radiation (TR), corticospinal tract (CST), IFOF, inferior longitudinal fasciculus (ILF), superior longitudinal fasciculus (SLF) [*p* < 0.05, false discovery rate (FDR) correction], and the pointwise analyses performed more sensitive. Furthermore, the negative correlations among MD, AD, RD, and symbol digit modalities test (SDMT) scores in the left TR (L_TR) were found in NMOSD.

**Conclusion:**

Patients with NMOSD exhibited the specific nodes of WM fiber tract damage, which can enhance our understanding of WM microstructural abnormalities in NMOSD. In addition, the altered DTI metrics were correlated with cognitive impairment, which can be used as imaging markers for the early identification of NMOSD cognitive impairment.

## Introduction

Neuromyelitis optica spectrum disorder (NMOSD) is an autoimmune inflammatory disease characterized by severe episodes of optic neuritis (ON), long-segment transverse myelitis (LETM), and area postrema syndrome (APS). It is mediated by perivascular immunoglobulin deposition and complement activation against the aquaporin-4 (AQP4) water channel in the central nervous system (Wingerchuk et al., 2007). With the deep understanding of NMOSD, 57–67% of NMOSD patients are accompanied by cognitive impairment, which mainly affects the prognosis of the disease (Saji et al., 2013; Liu et al., 2015; Moore et al., 2016).

Most neuroimaging studies of NMOSD have reported that structural damage predominantly restricted to white matter (WM) by diffusion tensor imaging (DTI) technology (Liu et al., 2012; Kimura et al., 2014; Pache et al., 2016; Kim et al., 2017; Cacciaguerra et al., 2021; Chen et al., 2021). Previous studies based on DTI mainly used voxel-based morphometry (VBM) and tract-based spatial statistics (TBSS) aimed at the voxel level but had the disadvantage of inaccurate positioning (Jones et al., 2005; Yeatman et al., 2012), and a few studies focused on the entire fiber tracts to analyze DTI parameters in patients with NMOSD (Kimura et al., 2014; Kuchling et al., 2018). However, it was known that only one segment of WM fiber tracts in patients with mild cognitive impairment (MCI) was destroyed with the other parts intact, and in the process of Alzheimer’s disease, the destruction of some WM fiber tracts follows a specific pattern (Zhang et al., 2019). Same as a neurological disease, we speculate that specific segments of WM fiber tract damage were similar in patients with NMOSD.

Automated fiber-tract quantification (AFQ) is an advanced DTI method for automated reconstruction of WM fiber tracts and segmentation of the fiber tracts into multiple nodes. It can provide more detailed information on WM damage and has been applied to MCI, schizophrenia, cerebral small vessel disease, benign childhood epilepsy, end-stage renal disease, and so on (Sun et al., 2015; Chen et al., 2020; Jiang et al., 2021; Shu et al., 2021; Stone et al., 2021). To our knowledge, the application of AFQ for patients with NMOSD has not been studied.

In this present study, the AFQ has been applied to (1) study the damage patterns of different 100 nodes of 20 specific cerebral WM fiber tracts in patients with NMOSD and (2) explore the correlations between the changes of DTI metrics and clinical cognitive performance.

## Materials and Methods

### Participants

This retrospective study has been approved by the Institutional Review Board of the First Affiliated Hospital of Chongqing Medical University, Chongqing, China, and written informed consent was obtained from each participant before magnetic resonance imaging (MRI) scans. In addition, demographic data and neuropsychological scales of the participants, such as gender, age, education level, disease duration, Hamilton depression (HAMD) scale, Hamilton anxiety (HAMA) scale, fatigue severity scale (FSS), digit span test (DST), symbol digit modalities test (SDMT), minimum mental state examination (MMSE), and Montreal Cognitive Assessment (MoCA) scores were also collected.

A total of 40 patients diagnosed with NMOSD and 20 sex-, age-, and education level-matched healthy controls (HCs) were enrolled between March 2021 and March 2022. Patients have received a confirmed diagnosis of NMOSD based on the standard diagnosis criteria (Wingerchuk et al., 2015), which has been free from relapse and not on the treatment of disease-modified medications within 4 weeks before MRI scans. All patients with NMOSD were serum positive for the AQP4 antibody. The exclusion criteria were (1) significant neurologic disease other than NMOSD; (2) image artifacts or incomplete clinical information; and (3) contraindications for MRI scans.

### Magnetic Resonance Imaging Acquisition

All MR scans were performed on a 3.0T MR scanner (Magnetom Skyra, Siemens Healthcare GmbH, Erlangen, Germany) using a 32-channel head coil. The MRI protocol included a 3-dimensional (3D) T_1_-weighted magnetization prepared rapid gradient echo (MPRAGE) sequence: echo time (TE) = 2.26 ms, repetition time (TR) = 2,300 ms, inversion time (TI) = 900 ms, 192 slices, field of view (FOV) = 256 mm, voxel size = 1.0 mm × 1.0 mm × 1.0 mm, and acquisition time (TA) = 5:21 min. In addition, a diffusion kurtosis imaging (DKI) sequence: TE = 97 ms, TR = 5,100 ms, 72 slices, FOV = 220 mm, voxel size = 1.7 mm × 1.7 mm × 2.0 mm, TA = 6:13 min, Parallel Acquisition Techniques (PATs) acceleration factor = 4, and three *b*-values (0, 1,000, and 2000) with diffusion encoding in 30 directions.

### Data Pre-processing

All DTI data were obtained by retaining *b*-values of 0 and 1,000 for DKI data and converting the data format by using the MRIcron software.^1^ Then the DTI images were preprocessed by using FMRIB Software Library (FSL) software,^2^ which included the following steps: (1) correction of subject motion, eddy-currents, and susceptibility-induced distortions; (2) generation of individual brain masks based on the B0 images of each subject by using a brain extraction tool (BET); (3) calculation of DTI matrix by using DTIFIT command of FSL to obtain fractional anisotropy (FA), mean diffusion (MD), axial diffusivity (AD), radial diffusivity (RD), eigenvalues (λ1, λ2, and λ3), eigenvectors (L1, L2, and L3), and a raw T_2_ image without diffusion weighting (S0).

For the 3D-T_1_ images, the pre-processing procedure included the following steps: (1) removal of non-brain structures by using BET; (2) calibration of the anterior commissure-posterior commissure (AC-PC) plane by using mrAnatAverageAcpcNifti function for subsequent alignment in VistaSoft package^3^; (3) alignment of T_1_ images to S0 images and transition of the DTI data to the format, which was fit for AFQ processing by using dtiMakeDt6FromFsl script in MATLAB R2012b.

### Automated Fiber Quantification

The open-source MATLAB version of AFQ software^4^ was applied to identify and quantify 100 equally spaced nodes of 20 specific WM fiber tracts for each participant (Yeatman et al., 2012). The entire processing procedure steps were as follows: (1) whole-brain deterministic fiber tractography with thresholds of FA > 0.2, turning angle < 30°, and fiber length between 50 and 250 mm; (2) waypoint regions of interest (ROIs)-based tract segmentation; (3) fiber tracts refinement based on probability maps; (4) fiber tracts cleaning into a compact tract spanning between cortical regions; and (5) fiber tracts quantification by clipping each fiber in the fiber group to the portion that spans between the two waypoint ROIs and resampling each fiber to 100 nodes.

**FIGURE 1:**
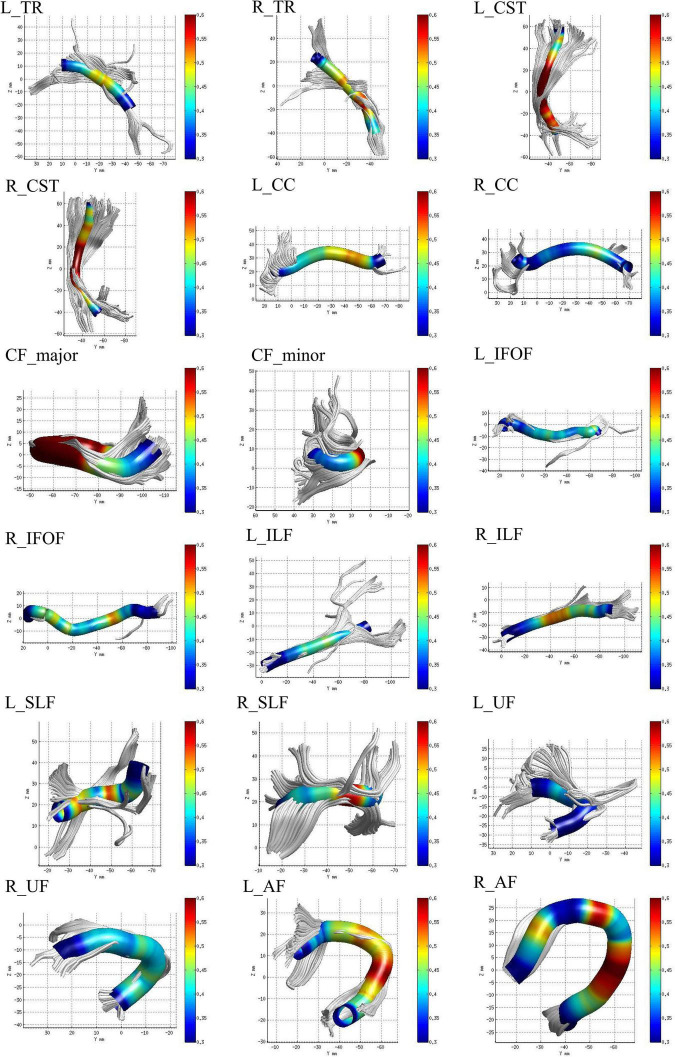
The 18 identified white matter (WM) fiber tracts, such as bilateral thalamic radiation (TR), corticospinal tract (CST), cingulum cingulate (CC), inferior fronto-occipital fasciculus (IFOF), inferior longitudinal fasciculus (ILF), superior longitudinal fasciculus (SLF), uncinate fasciculus (UF), arcuate fasciculus (AF), callosum forceps major (CF_major), and minor (CF_minor) by automated fiber-tract quantification (AFQ).

The identified 20 WM fiber tracts included bilateral thalamic radiation (TR), corticospinal tract (CST), cingulum cingulate (CC), cingulum hippocampus (CH), inferior fronto-occipital fasciculus (IFOF), inferior longitudinal fasciculus (ILF), superior longitudinal fasciculus (SLF), uncinate fasciculus (UF), arcuate fasciculus (AF), callosum forceps major (CF_major), and callosum forceps minor (CF_minor). However, there were nearly half of the subjects who failed to identify bilateral CH fiber tracts due to the AFQ’s strict criteria for fiber tract identification (Table 1). Therefore, we excluded the two fiber tracts and enrolled the other 18 fiber tracts in our study for further analysis. The 18 identified WM fiber tracts are showed in Figure 1.

**TABLE 1:** Identification number and ratio for 20 WM fiber tracts in NMOSD and HC.

Tracts	Number of participants			Ratio of participants		
	NMOSD	HC	Total	NMOSD (%)	HC (%)	Total (%)
L_TR	40	20	60	100.0	100.0	100.0
R_TR	40	20	60	100.0	100.0	100.0
L_CST	40	20	60	100.0	100.0	100.0
R_CST	40	20	60	100.0	100.0	100.0
L_CC	40	20	60	100.0	100.0	100.0
R_CC	40	20	60	100.0	100.0	100.0
L_CH	24	12	36	60.0	60.0	60.0
R_CH	23	17	40	57.5	85.0	66.7
CF_major	39	20	59	97.5	100.0	98.3
CF_minor	40	20	60	100.0	100.0	100.0
L_IFOF	40	20	60	100.0	100.0	100.0
R_IFOF	40	20	60	100.0	100.0	100.0
L_ILF	39	20	59	97.5	100.0	98.3
R_ILF	40	20	60	100.0	100.0	100.0
L_SLF	40	20	60	100.0	100.0	100.0
R_SLF	40	20	60	100.0	100.0	100.0
L_UF	40	20	60	100.0	100.0	100.0
R_UF	40	20	60	100.0	100.0	100.0
L_AF	39	20	59	97.5	100.0	98.3
R_AF	36	20	56	90.0	100.0	93.3

*NMOSD, neuromyelitis optica spectrum disorder; HC, healthy controls; TR, thalamic radiation; CST, corticospinal tract; CC, cingulum cingulate; CH, cingulum hippocampus; CF_major, callosum forceps major; CF_minor, callosum forceps minor; IFOF, inferior fronto-occipital fasciculus; ILF, inferior longitudinal fasciculus; SLF, superior longitudinal fasciculus; UF, uncinate fasciculus; AF, arcuate fasciculus; L, left; R, right.*

### Statistical Analysis

All statistical analyses, such as demographic data and neuropsychological scales, were performed by using the SPSS software (version 25.0; SPSS, Chicago, IL, United States). The normal distribution of the data was assessed by using the Kolmogorov-Smirnov test. According to the data whether normally distributed, independent samples *t*-tests or Mann–Whitney U tests were used to assess between-group differences. The gender of the subjects was analyzed using Pearson’s Chi-square tests. *p* < 0.05 was considered statistically significant.

The DTI metrics, such as FA, MD, AD, and RD, at each node along the 18 identified WM fiber tracts were compared between NMOSD and HCs in entire and pointwise manners using independent samples *t*-tests. All statistical results were considered significant when *p* < 0.05 after false discovery rate (FDR) correction for multiple comparisons. In a pointwise manner, only significant differences observed at ≥ 3 adjacent nodes were reported (Banfi et al., 2019).

The further Pearson or Spearman correlation analyses were performed between the neuropsychological scales and mean DTI metrics of WM fiber clusters or entire fiber tracts where significant differences were investigated in patients with NMOSD. After FDR correction, *p*-value < 0.05 represents the significant difference.

## Results

### Demographic Data and Neuropsychological Scales

The demographic data and neuropsychological scales of participants are summarized in Table 2. There were no significant differences in gender, age, and education level between NMOSD and HCs. However, when compared to HCs, NMOSD showed higher HAMD, HAMA, FSS, and lower DST, SDMT, MMSE, MoCA scores (*p* < 0.05).

**TABLE 2:** Demographic data and neuropsychological scales of participants.

Characteristics	NMOSD (*n* = 40)	HC (*n* = 20)	*t*/*Z*/χ*^2^*-value	*P*-value
Gender (female/male)	37/3	18/2	0.109	0.741^a^
Age (years)	39.80 ± 12.54	39.15 ± 8.56	0.208	0.836^b^
Education level (years)	12.00 (9.75, 15.00)	15.00 (9.75, 16.00)	–1.266	0.205^c^
Disease duration	3.80 (2.30, 6.90)	–	–	–
HAMD	6.00 ± 4.72	0.00 (0.00, 1.00)	5.136	< 0.001^c^,*
HAMA	5.00 (1.75, 10.00)	0.00 (0.00, 1.00)	5.113	< 0.001*y*^c^,*
FSS	33.05 ± 14.25	12.50 (9.25, 23.25)	4.357	< 0.001^c^,*
DST	14.08 ± 2.94	16.20 ± 2.42	–2.764	0.008^b^,*
SDMT	42.47 ± 16.76	54.20 ± 15.19	–2.568	0.013^b^,*
MMSE	28.00 (27.00, 29.00)	29.00 (28.25, 30.00)	–2.585	0.010^c^,*
MoCA	24.00 (22.00, 28.00)	28.00 (27.00, 29.00)	–3.382	0.001^c^,*

*HAMD, Hamilton depression scale; HAMA, Hamilton anxiety scale; FSS, fatigue severity scale; DST, digit span test; SDMT, symbol digit modalities test; MMSE, minimum mental state examination; MoCA, Montreal Cognitive Assessment.*

*^a^p and χ^2^values were obtained using the Pearson’s Chi-square tests.*

*^b^p and t-values were obtained using the independent sample t-tests.*

*^c^p and Z-values were obtained using the Mann-Whitney U tests.*

*p-value < 0.05 represents the significant difference.

### Alterations in Entire White Matter Fiber Tracts

For FA of the entire WM fiber tracts, as compared to HC, NMOSD showed a significant reduction in the L_IFOF (*t* = –2.254, *p* = 0.026).

For MD of the entire WM fiber tracts, NMOSD showed a significant increment in the L_TR (*t* = 2.388, *p* = 0.020), L_CST (*t* = 4.403, *p* < 0.001), R_CST (*t* = 3.359, *p* = 0.001), R_CC (*t* = 2.892, *p* = 0.005), L_IFOF (*t* = 2.226, *p* = 0.030), R_IFOF (*t* = 3.370, *p* = 0.001), L_ILF (*t* = 2.081, *p* = 0.042), L_SLF (*t* = 3.361, *p* = 0.001), and R_AF (*t* = 2.408, *p* = 0.019).

For AD of the entire WM fiber tracts, NMOSD showed a significant increment in the L_TR (*t* = 2.606, *p* = 0.012), L_CST (*t* = 2.442, *p* = 0.018), R_CST (*t* = 2.551, *p* = 0.013), R_CC (*t* = 3.040, *p* = 0.004), R_IFOF (*t* = 2.705, *p* = 0.009), L_SLF (*t* = 2.768, *p* = 0.008), R_UF (*t* = 2.431, *p* = 0.018), and L_AF (*t* = 3.420, *p* = 0.001).

**FIGURE 2:**
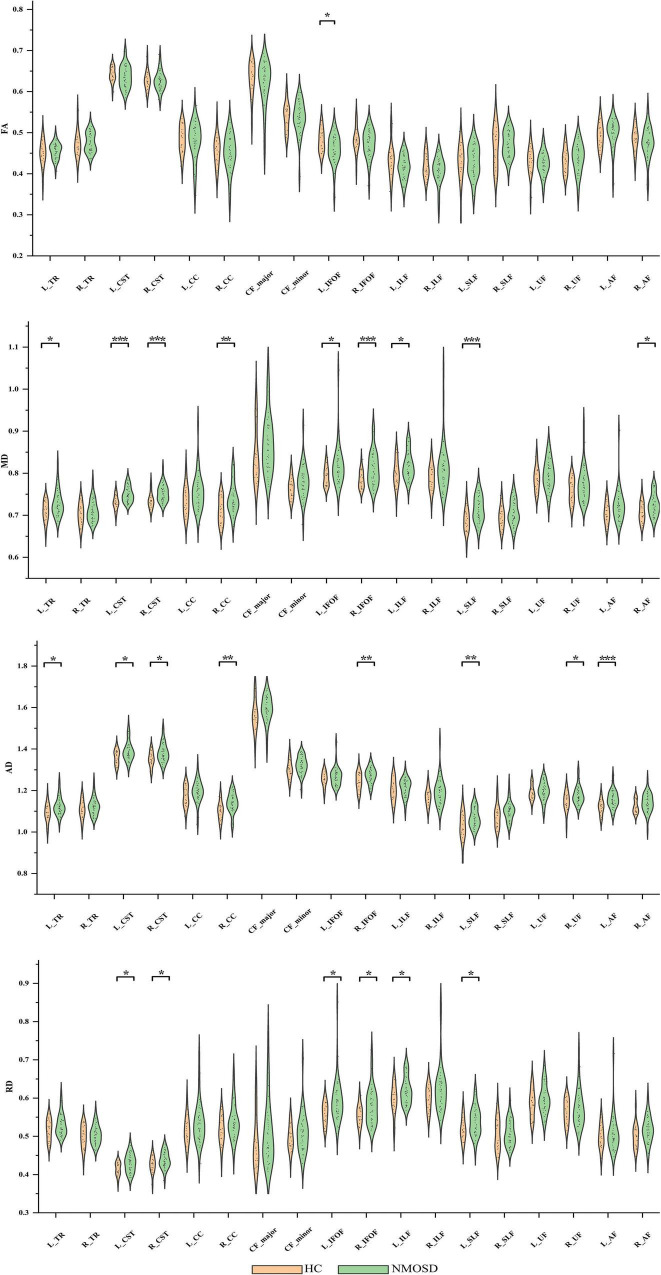
Group differences in entire white matter (WM) fiber tracts of diffusion tensor imaging (DTI) metrics, such as fractional anisotropy (FA), mean diffusion (MD), axial diffusivity (AD), and radial diffusivity (RD). *p < 0.05, **p < 0.01, ***p < 0.001. NMOSD, neuromyelitis optica spectrum disorder; HC, healthy controls.

For RD of the entire WM fiber tracts, NMOSD showed a significant increment in the L_CST (*t* = 2.515, *p* = 0.015), R_CST (*t* = 2.054, *p* = 0.044), L_IFOF (*t* = 2.435, *p* = 0.018), R_IFOF (*t* = 2.592, *p* = 0.012), L_ILF (*t* = 2.243, *p* = 0.029), and L_SLF (*t* = 2.234, *p* = 0.029). All group differences in entire WM fiber tracts of DTI metrics, such as FA, AD, MD, and RD, are showed in Figure 2.

### Alterations in Pointwise White Matter Fiber Tracts

**FIGURE 3:**
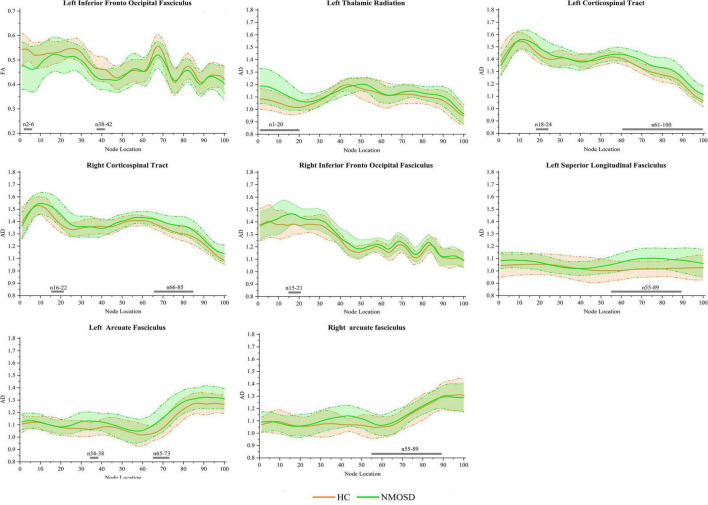
Group differences in pointwise white matter (WM) fiber tracts of fractional anisotropy (FA) and axial diffusivity (AD) between neuromyelitis optica spectrum disorder (NMOSD) and healthy controls [HCs; *p* < 0.05, false discovery rate (FDR) correction]. The green line represents the NMOSD group, and the orange line represents the HC group [solid lines for means and shaded regions for standard deviations (SDs)]. The gray bars at the bottom are the location of the fiber nodes with significant differences between the two groups.

In the pointwise WM fiber tracts comparison of FA, when compared to HCs, NMOSD showed a significant reduction in the anterior portion of the L_IFOF (nodes 2–6 and 38–42; Figure 3, *p* < 0.05, FDR correction).

In the pointwise WM fiber tracts comparison of AD, NMOSD showed a significant increment in widespread positions as follows: (1) the anterior portion of the L_TR (nodes 1–20); (2) the anterior and posterior portions of the L_CST (nodes 18–24 and 61–100); (3) the anterior and posterior portions of the R_CST (nodes 16–22 and 66–85); (4) the anterior portion of the R_IFOF (nodes 15–21); (5) the posterior portion of the L_SLF (nodes 55–89); (6) the anterior and posterior portions of the L_AF (nodes 34–38 and 65–73); and (7) the posterior portion of the R_AF (nodes 55–89; Figure 3, *p* < 0.05, FDR correction).

**FIGURE 4:**
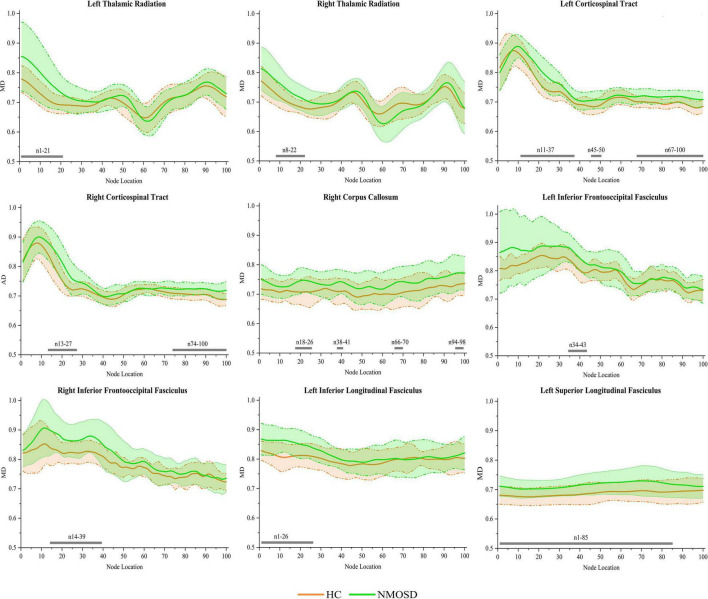
Group differences in pointwise white matter (WM) fiber tracts of mean diffusion (MD) between NMOSD and healthy control [HC; *p* < 0.05, false discovery rate (FDR) correction]. The green line represents the NMOSD group, and the orange line represents the HC group (solid lines for means and shaded regions for standard deviations). The gray bars at the bottom are the location of the fiber nodes with significant differences between the two groups.

In the pointwise WM fiber tract comparison of MD, NMOSD showed a significant increment in widespread positions as follows: (1) the anterior portion of the L_TR (nodes 1–21); (2) the anterior portion of the R_TR (nodes 8–22); (3) the anterior and posterior portions of the L_CST (nodes 11–37, 45–50, and 67–100); (4) the anterior and posterior portions of the R_CST (nodes 13–27 and 74–100); (5) the anterior and posterior portions of the R_CC (nodes 18–26, 38–41, 66–70, and 94–98); (6) the anterior portion of the L_IFOF (nodes 34–43); (7) the anterior portion of the R_IFOF (nodes 14–39); (8) the anterior portion of the L_ILF (nodes 1–26); and (9) almost entire portion of the L_SLF (nodes 1–85; Figure 4, *p* < 0.05, FDR correction).

**FIGURE 5:**
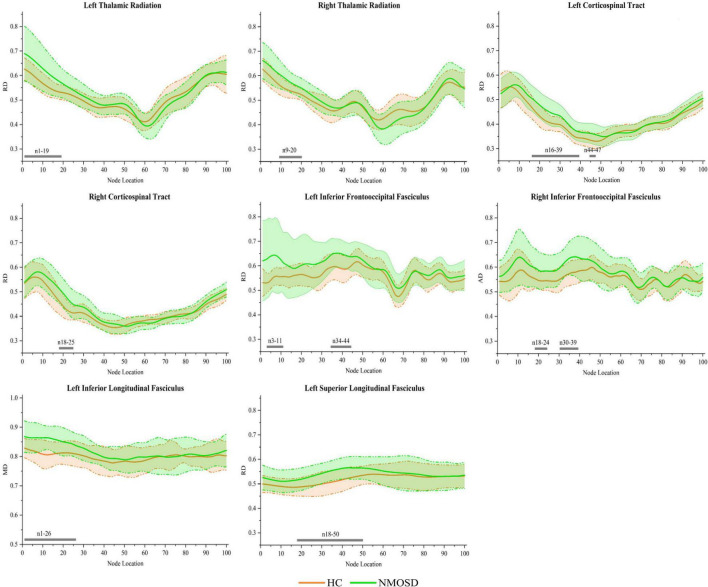
Group differences in pointwise white matter (WM) fiber tracts of radial diffusivity (RD) between neuromyelitis optica spectrum disorder (NMOSD) and healthy control [HC; *p* < 0.05, false discovery rate (FDR) correction]. The green line represents the NMOSD group, and the orange line represents the HC group (solid lines for means and shaded regions for standard deviations). The gray bars at the bottom are the location of the fiber nodes with significant differences between the two groups.

In the pointwise WM fiber tract comparison of RD, NMOSD showed a significant increment in widespread positions as follows: (1) the anterior portion of the L_TR (nodes 1–19); (2) the anterior portion of the R_TR (nodes 9–20); (3) the anterior portion of the L_CST (nodes 16–39 and 44–47); (4) the anterior portion of the R_CST (nodes 18–25); (5) the anterior portion of the L_IFOF (nodes 3–11 and 34–44); (6) the anterior portion of the R_IFOF (nodes 18–24 and 30–39); (7) the anterior portion of the L_ILF (nodes 1–16, 23–25, and 32–38); and (8) the anterior portion of the L_SLF (nodes 18–50; Figure 5, *p* < 0.05, FDR correction).

### Correlations Between Diffusion Tensor Imaging Metrics and Clinical Cognitive Performance

**FIGURE 6:**
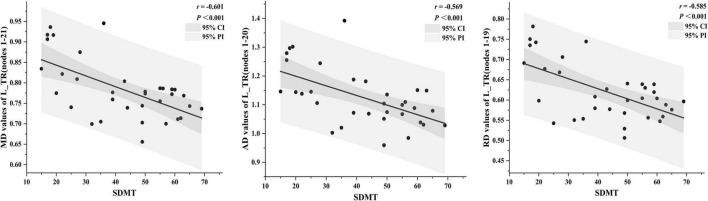
The correlations between diffusion tensor imaging (DTI) metrics and clinical cognitive performance [*p* < 0.05, false discovery rate (FDR) correction]. The dark gray region represents the 95% confidence interval (CI), and the light gray region represents the 95% prediction interval (PI). SDMT, symbol digit modalities test.

Mean diffusion in nodes 1–21 of the L_TR (*r* = –0.601, *p* < 0.001), AD in nodes 1–20 of the L_TR (*r* = –0.569, *p* < 0.001), and RD in nodes 1–19 of the L_TR (*r* = –0.585, *p* < 0.001) had negative correlations with the SDMT scores (Figure 6, *p* < 0.05, FDR correction).

However, no significant correlations between DTI metrics and the other neuropsychological scales, such as HAMA, HAMD, FSS, DST, MMSE, and MoCA, were observed in patients with NMOSD (*p* > 0.05, FDR correction).

## Discussion

In the present study, we investigated the alterations of the tract-specific WM integrity in patients with NMOSD by AFQ and their correlations with cognitive impairment. We found that patients with NMOSD had an extensive WM fiber tract microstructural damage not only at the entire but also at the pointwise level, and the pointwise analyses were performed more sensitively. Furthermore, the DTI metrics, such as MD, AD, and RD, extracted from L_TR showed negative correlations with cognitive impairment.

Diffusion tensor imaging technology is a powerful non-invasive tool for studying complex brain tissue structures. The DTI metrics, such as FA, MD, AD, and RD, are highly sensitive to detect microstructural characteristics, such as fiber density, fiber coherence, fiber orientation, degree of myelination, and WM integrity. In addition, the AFQ is an ideal algorithm based on DTI, which can provide more precise information on the fiber tracts than VBM, TBSS, and traditional tractography. It has been applied to various WM damage-related diseases but NMOSD.

In the current study, for the entire WM fiber tracts, as compared to HC, patients with NMOSD exhibited a decreased FA in the L_IFOF and increased MD, AD, and RD in a wide range of WM fiber tracts, which indicated widespread diffusion changes in patients with NMOSD. It was in agreement with some recent research studies (Kim et al., 2017; Cacciaguerra et al., 2021). However, as reported in several previous studies, only the FA reduction was found in patients with NMOSD, which indicated that the integrity of axons had been damaged, but the extent of the damage may still be relatively minor (Jeong et al., 2016; Chen et al., 2021). It may be due to the longer disease duration and the more lesions contribution of patients included in this study, resulting in more severe WM damage, which can be confirmed by comparison with another research (Kim et al., 2017). It showed the patients with NMOSD had significantly lower mean global FA, higher MD and RD in many WM tracts based on TBSS and ROIs analysis, which was similar to ours. FA reflects the ratio of anisotropic components of water molecules to the entire diffusion tensor, indirectly reflecting the integrity of WM fiber tracts; MD value reflects the degree of diffusion of all water molecules in all directions, showing the diffusion ability of water molecules in tissues; AD reflects the diffusion of water molecules parallel to the direction of nerve fibers, which can provide information on axonal integrity. RD reflects the diffusion of water molecules perpendicular to the direction of nerve fibers, which can provide information about the number of myelin sheaths around nerve cells (Le Bihan et al., 2001; Assaf and Pasternak, 2008). Thus, the reduced FA in the L_IFOF and widespread increased MD, AD, and RD may reflect diffused abnormalities of myelin and fiber axons to cause the widespread WM damage in patients with NMOSD. Interestingly, based on the results, we speculate that MD, AD, and RD seemed to be more sensitive to WM damage than FA, consistent with the previous studies (Jin et al., 2017; Chen et al., 2020). It is worth noting that based on the whole WM fiber tracts by previous TBSS or ROI analysis, it just found out the abnormality of the fiber tracts, its specific regional abnormality was not clear, but the pointwise comparative analysis by AFQ can make up for this defect.

In the pointwise comparison of WM fiber tracts, not only the damaged segments of the different tracts that were more accurately detected but also a few segments of the tracts without differences were also shown. It proved our hypothesis that the destruction of some WM fiber tracts in NMOSD followed a specific pattern. It was also noteworthy that regardless of the entire or pointwise level, the results showed a widespread WM damage, especially for the TR, projection fiber (CST), and association fibers (IFOF, ILF, and SLF), which indicated that the extent of the above WM fiber tract damage may be relatively greater. The TR connects the thalamus with the cerebral hemispheres, while the damage occurred in the thalamus whether in the acute or stable phase of NMOSD (Zhao et al., 2012). The CST is a collection of axons that carry movement-related information from the cerebral cortex to the spinal cord. In addition, the IFOF, ILF, and SLF are the long associative tracts of neurons in the human brain, and it connects various parts, which makes them more vulnerable than other tracts (Chen et al., 2020). These results were similar to several previous TBSS studies explained by the WM secondary degeneration that occurred owing to ON and myelitis (Rueda Lopes et al., 2012; Kim et al., 2017), however, our study can identify more accurate damage location by AFQ.

Similar to the prior studies that reported cognitive impairment in NMOSD, our study showed that NMOSD had higher HAMD, HAMA, and FSS and lower DST, SDMT, MMSE, and MoCA scores than HC (Pache et al., 2016; Zheng et al., 2021). In addition, the correlation analyses were performed between the neuropsychological scales and mean DTI metrics of WM fiber clusters or entire fiber tracts where significant differences were investigated in patients with NMOSD. After FDR correction, our results showed that the SDMT scores had negative correlations with MD, AD, and RD in the anterior portion of the L_TR, which suggests that the more serious the damage to WM microstructure, the worse is the cognitive performance. The TR connects the mediodorsal and anterior thalamus to the prefrontal cortex and the anterior cingulate cortex, and the thalamus is a complex structure, which affects the most sensory and motor pathways and acts as a source of information for the frontal and motor areas (Setiadi et al., 2021). Therefore, the damage to TR may be related to the cognitive impairment. In addition, the correlations were only found from TR in the left hemisphere. The parts of the reasons for the discovery were that (1) the WM vulnerability in NMOSD may have hemispheric heterogeneity similar to Alzheimer’s disease (Chen et al., 2020), and (2) the SDMT is widely used to assess cognitive performance, such as divided attention, perceptual processing speed, visual scanning, and memory. It may mainly be affected by the left hemisphere, which is the abstract thinking center. In conclusion, L_TR played an important role in the cognitive performance of patients with NMOSD, which can be used as imaging markers for the early identification of NMOSD cognitive impairment.

Several limitations also exist in this work. First, only a central portion of the fiber tract is analyzed by AFQ. Although in this way, we will miss some marginal information, it can avoid the need for additional coregistration procedures. Second, due to the AFQ’s strict criteria for fiber tract identification, we could not identify bilateral CH fiber tracts. Finally, the sample size was relatively small. Future work was required to increase the sample size to validate our results.

## Conclusion

In conclusion, this study identified specific alterations of 18 WM fiber tracts using the AFQ approach, which will enhance our understanding of WM microstructural abnormalities in NMOSD. In addition, the correlations between DTI altered metrics and cognitive impairment in NMOSD can be used as imaging markers for early identification of NMOSD cognitive impairment.

## Data Availability Statement

The raw data supporting the conclusions of this article will be made available by the authors, without undue reservation.

## Ethics Statement

The studies involving human participants were reviewed and approved by the Institutional Review Board of the First Affiliated Hospital of Chongqing Medical University. The patients/participants provided their written informed consent to participate in this study.

## Author Contributions

ZY offered the research ideas, performed the data processing, and prepared the manuscript. XW performed the statistical analyses and revised the manuscript. QZu performed the graphing. XC assisted in the methodology. ZS offered the data collection support. YH and QZe assisted in MRI scanning. YW provided the revision. JW and YL provided guidance and critical reviews. All authors contributed to the article and approved the submitted version.

## Conflict of Interest

The authors declare that the research was conducted in the absence of any commercial or financial relationships that could be construed as a potential conflict of interest.

## Publisher’s Note

All claims expressed in this article are solely those of the authors and do not necessarily represent those of their affiliated organizations, or those of the publisher, the editors and the reviewers. Any product that may be evaluated in this article, or claim that may be made by its manufacturer, is not guaranteed or endorsed by the publisher.

## Footnotes

1
http://www.mricro.com

2
https://fsl.fmrib.ox.ac.uk/fsl/fslwiki/

3
https://github.com/vistalab/vistasoft

4
https://github.com/jyeatman/AFQ

## Abbreviations

NMOSD: neuromyelitis optica spectrum disorder
WM: white matter
DTI: diffusion tensor imaging
AFQ: automated fiber-tract quantification
FA: fractional anisotropy
MD: mean diffusion
AD: axial diffusivity
RD: radial diffusivity
TR: thalamic radiation
CST: corticospinal tract
CC: cingulum cingulate
CH: cingulum hippocampus
IFOF: inferior fronto-occipital fasciculus
ILF: inferior longitudinal fasciculus
SLF: superior longitudinal fasciculus
UF: uncinate fasciculus
AF: arcuate fasciculus
CF_major: callosum forceps major
CF_minor: callosum forceps minor.
